# Intramuscular Ganglion Cyst of the Extensor Digitorum Longus

**DOI:** 10.1155/cro/8881015

**Published:** 2026-04-01

**Authors:** Ramy Saade, Ahmad Hammad, Jean Paul Rizk, Najla Fakhruddin, Mohamad Nassereddine

**Affiliations:** ^1^ Department of Orthopedics Surgery, American University of Beirut Medical Center, Beirut, Beirut Governate, Lebanon, aubmc.org.lb; ^2^ Department of Pathology, American University of Beirut Medical Center, Beirut, Beirut Governate, Lebanon, aubmc.org.lb

**Keywords:** ganglion cyst, intramuscular ganglion cyst, soft tissue tumor, surgical excision

## Abstract

**Background:**

Ganglion cyst is a mucin‐filled synovial cyst. It is the most common mass found in the wrist and hand. However, it is less commonly encountered in the lower extremity and even rarer to be found intramuscularly.

**Case Presentation:**

A 34‐year‐old female presented for a symptomatic mass at the anterolateral left knee. Radiography revealed an intramuscular multiseptated cystic lesion within the extensor digitorum longus muscle. No definite solid components or enhancement of the lesion and no intra‐articular extension or capsular communication were observed. Differential diagnosis included intraosseous myxoma and atypical ganglion cyst with inability to exclude malignancy. The patient underwent an open excisional biopsy that corresponded to a ganglion cyst on histopathological assessment.

**Conclusions:**

Intramuscular ganglion cysts are rare and underreported in the literature. A thorough differential diagnosis for lesions in uncommon anatomical locations is essential. Surgical excision with histological confirmation remains crucial for diagnostic and therapeutic purposes to rule out malignancy and prevent recurrence.

## 1. Introduction

Ganglion cysts are benign cystic masses containing highly viscous, proteinaceous material, encapsulated by a dense fibrous connective tissue capsule [1]. It is postulated that these cysts arise from mesenchymal cells of the joint capsule or tendon sheath secondary to repetitive microinjury, which stimulates fibroblasts to produce hyaluronic acid within these cysts [2].

Ganglion cysts occur more commonly in the upper extremity, with around 70% found around the hand and wrist [2]. Cysts around the distal femur usually occur within the synovial membrane or tendon sheath but rarely within muscles [3]. Nonetheless, intramuscular ganglion cysts, that is, cysts with no connection to the knee joint, are even more rare [4].

Most ganglion cysts are asymptomatic; however, patients may present with pain, tenderness, weakness, or dissatisfaction with cosmetic appearance [3]. Due to this cyst′s proximity to the knee joint, x‐ray is the initially used imaging modality. This is followed by ultrasonography being the next and magnetic resonance imaging (MRI) reserved for atypical presentations or concerns for malignancy [5]. Treatment options can vary between conservative management with close monitoring and observation, to aspiration under fluoroscopic guidance, and eventually surgical excision and histopathologic assessment [3, 4].

The aim of this paper is to report on a rare case of intramuscular ganglion cyst of the extensor digitorum longus muscle that, to our knowledge, has been previously reported as intratendinous [6]. Both oral and written informed consents were obtained regarding the case study and future publications, and the work has been reported in line with the CARE checklist and criteria.

## 2. Case Presentation

**Figure 1:**
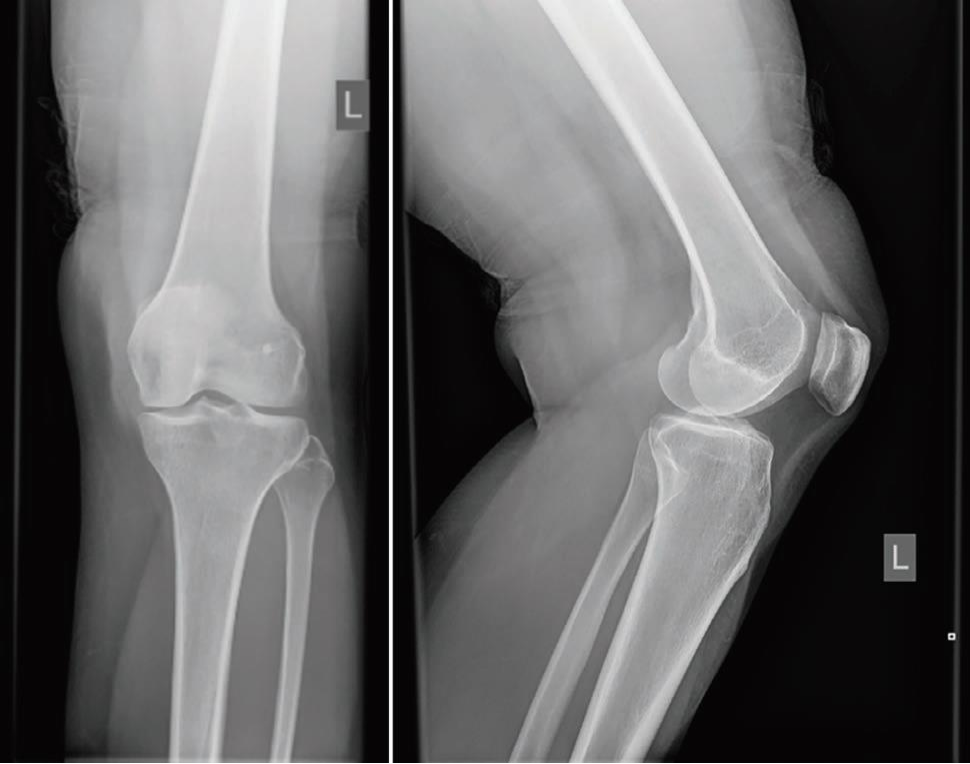
X‐ray of the knee without evidence of focal bone lesion or erosion or active periostitis and no significant knee joint effusion.

**Figure 2:**
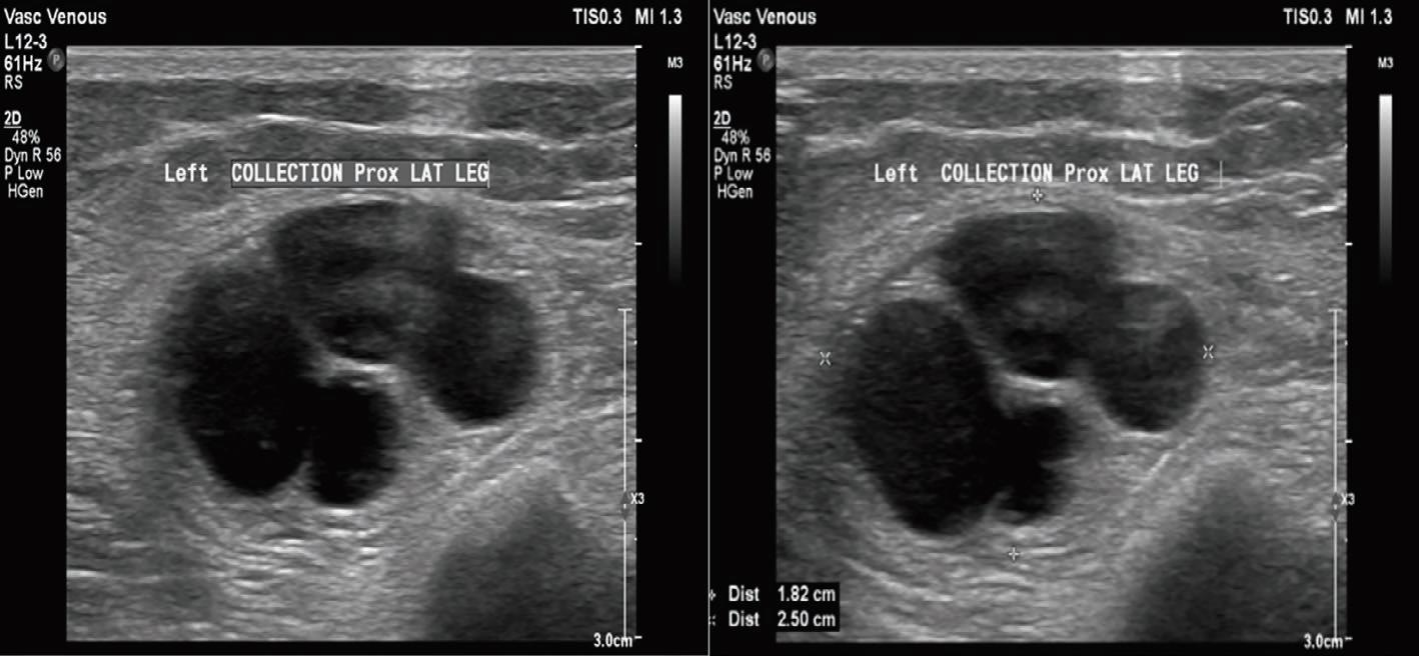
Ultrasound of the knee showing intramuscular complex tumor with cystic component and thick irregular septations measuring 7 × 2.6 × 2.1 cm.

A previously healthy 34‐year‐old female presented to the vascular clinic in July of 2024 for discomfort in the bilateral legs. She was diagnosed with varicose veins and treated accordingly. She also reported a left knee mass of several months causing pain and discomfort. She reports feeling a mass at the anterolateral aspect of the left leg associated with some pain below the knee joint line but without associated numbness or weakness of the lower extremity. On physical examination, she had a palpable hard mass at the inferior anterolateral aspect of the knee with full range of motion. She was originally investigated with x‐ray, which showed mild subchondral sclerosis and medial joint space narrowing suggestive of early osteoarthritic changes (Figure 1). She was referred to our orthopedic surgery clinics in August, where an ultrasound was ordered, showing a complex mass with a cystic component and thick irregular septations measuring 7 × 2.6 × 2.1 cm (Figure 2).

To complete her assessment and aid in establishing a diagnosis, an MRI was then performed revealing an intramuscular multiseptated soft tissue lesion with thin peripheral and septal enhancement. The lesion involved the extensor digitorum longus muscle abutting the anterior cortex of the fibular head and neck but not involving the common peroneal nerve (Figure 3).

Figure 3MRI bilateral lower extremity. (a) Coronal cut of knee showing intramuscular multiseptated lesion with thin peripheral and septalenhancement, (orange arrow). (b) Axial cut of lesion within the extensor digitorum longus abutting the anterior cortex of the fibular head and neckbut not communicating with the anterior tibiofibular joint capsule. TA, tibialis anterior; EHL, extensor hallucis longus; EDL, extensor digitorumlongus. (c) Coronal cut STIR sequence showing high signal intensity (blue arrow).

**Figure:**
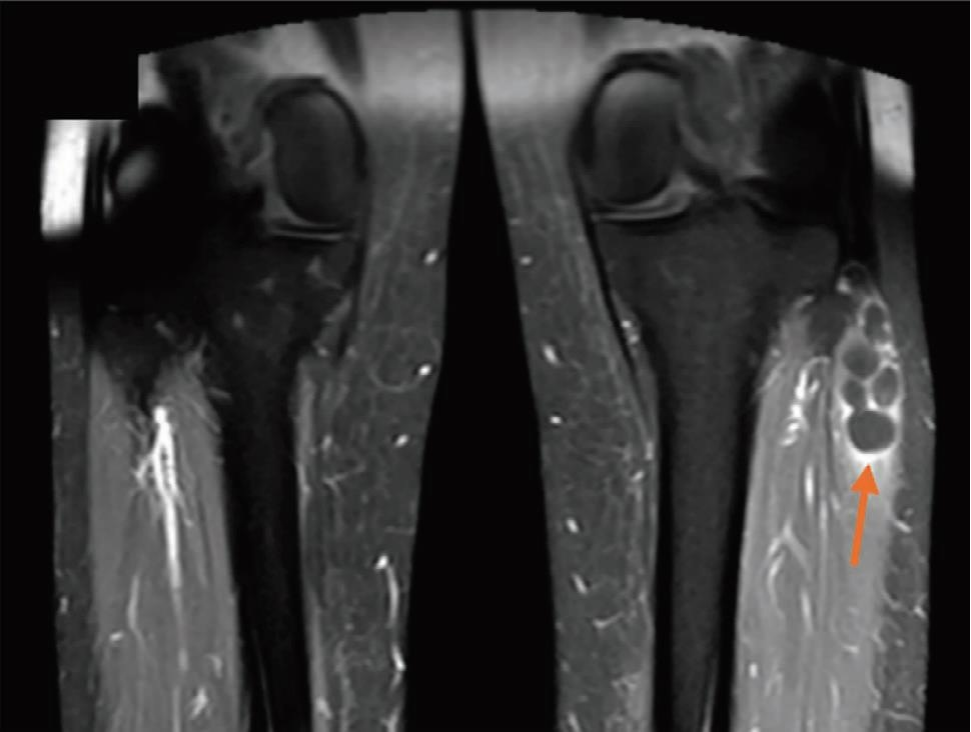
(a)

**Figure:**
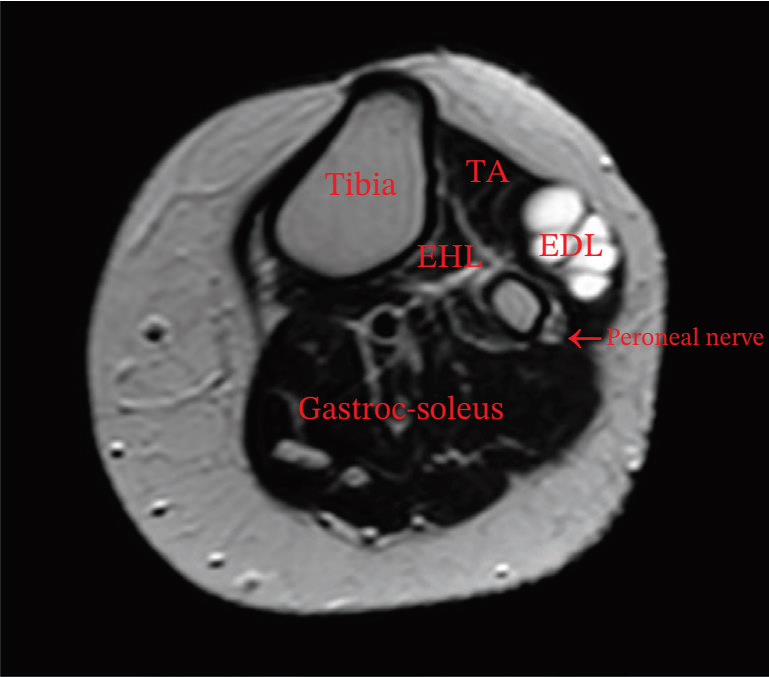
(b)

**Figure:**
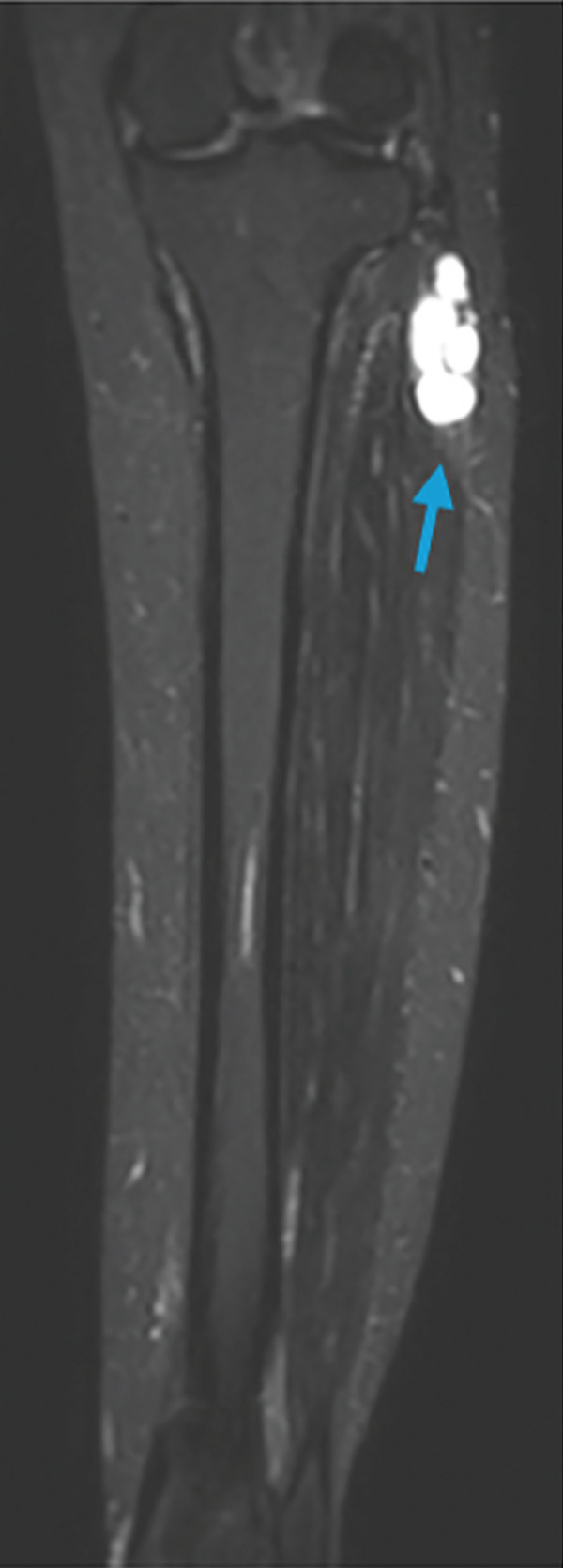
(c)

After discussion with the patient, a period of observation was agreed on. The patient re‐presented to clinic in February of 2025 with persistent symptoms of pain and discomfort and aesthetic concerns. Having given a period watchful waiting of nearly 6 months. The patient decided to proceed with an intervention. Percutaneous aspiration versus open excision was discussed. However, by mentioning the recurrence rate being up to 50% with aspiration, the patient opted for open excision in the operating room [7]. Open excisional biopsy would be both diagnostic and therapeutic with a success rate of up to 95% [8]. The lesion was not arising from or communicating with the anterior capsule of the proximal tibiofibular joint. Based on radiographic findings, differential diagnosis included intraosseous myxoma and atypical ganglion cyst with inability to exclude malignancy, such as myxoid liposarcoma and synovial sarcoma. The timeline is summarized in Table 1.

**Table 1:** Timeline of patient presentation and treatment.

Date	Diagnostic test	Result	Diagnosis
July 2024	X‐ray of the knee	Early osteoarthritis	Osteoarthritis
August 2024	Ultrasound of mass	Cystic mass with septations	Myxoma versus ganglion cyst
August 2024	MRI of the leg	Cystic lesion with septal enhancement	More in favor of ganglion cyst
February 2025	Open excisional biopsy	Atypical ganglion cyst	Intraosseous myxoma versus atypical ganglion cyst with inability to exclude malignancy

In view of the possibility of a malignant lesion that has been symptomatic for months and interfering with daily living and the inconclusiveness of the biopsy, decision was discussed with the patient to proceed with surgical resection in March of 2025. An anterolateral incision overlying the mass gave access to the lesion, which was found within the substance of the extensor digitorum longus muscle and without capsular violation. Given the proximity of the mass to the fibular head, the peroneal nerve was identified and protected. The mass was multinodular with jelly‐like contents and was then resected en bloc and sent to pathology. The patient had a smooth postoperative course without neurovascular deficit and was allowed immediate weight‐bearing as tolerated.

Further pathological and histological analysis was performed. Gross examination showed a fragment of skeletal muscle measuring 7 × 2.6 × 2 cm with a tan‐white cystic cut surface filled with mucoid material. Microscopically, the cyst is multilocular, surrounded by skeletal muscle and a layer of hypocellular connective tissue. It lacks an epithelial lining and contains a basophilic mucoid material without cytological atypia or mitotic activity (Figure 4). The bland morphological features support the diagnosis of ganglion cyst. In more cellular lesions, the main differential diagnosis to consider morphologically is low‐grade myxofibrosarcoma. However, the absence of cytological atypia and the lack of characteristic curvilinear blood vessels effectively excluded myxofibrosarcoma.

Figure 4(a) A cyst filled with basophilic mucoid material surrounded by skeletal muscle and connective tissue. (b) The hypocellular connective tissue and the absence of an epithelial lining.

**Figure:**
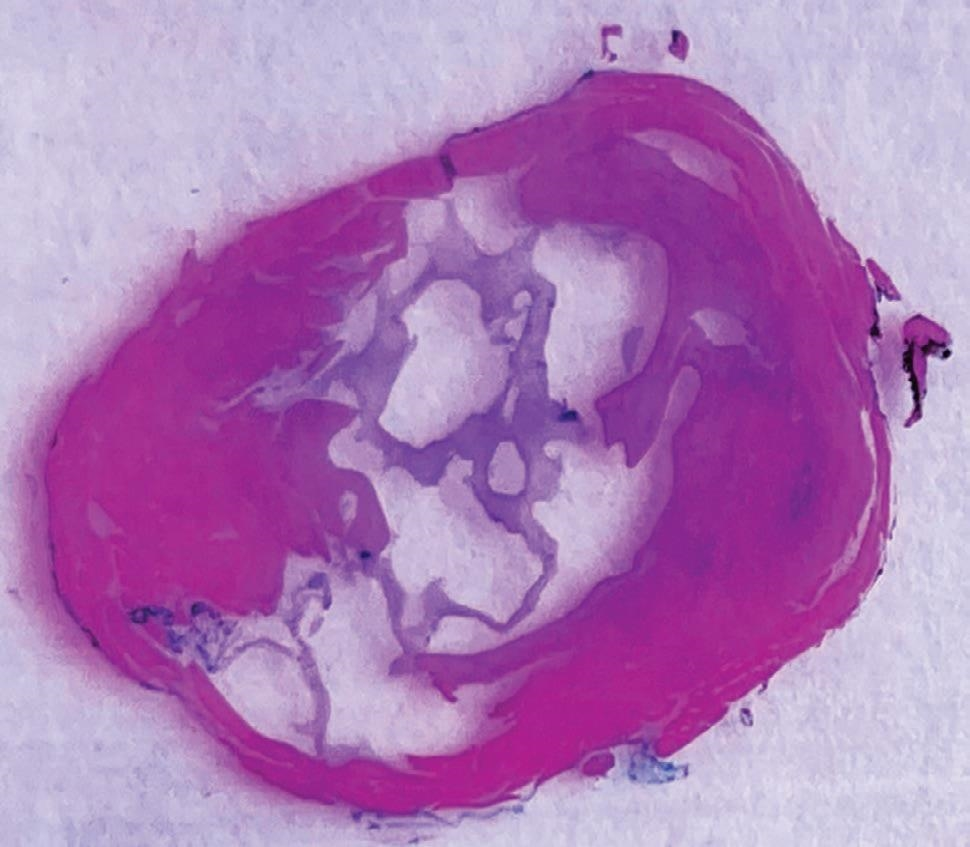
(a)

**Figure:**
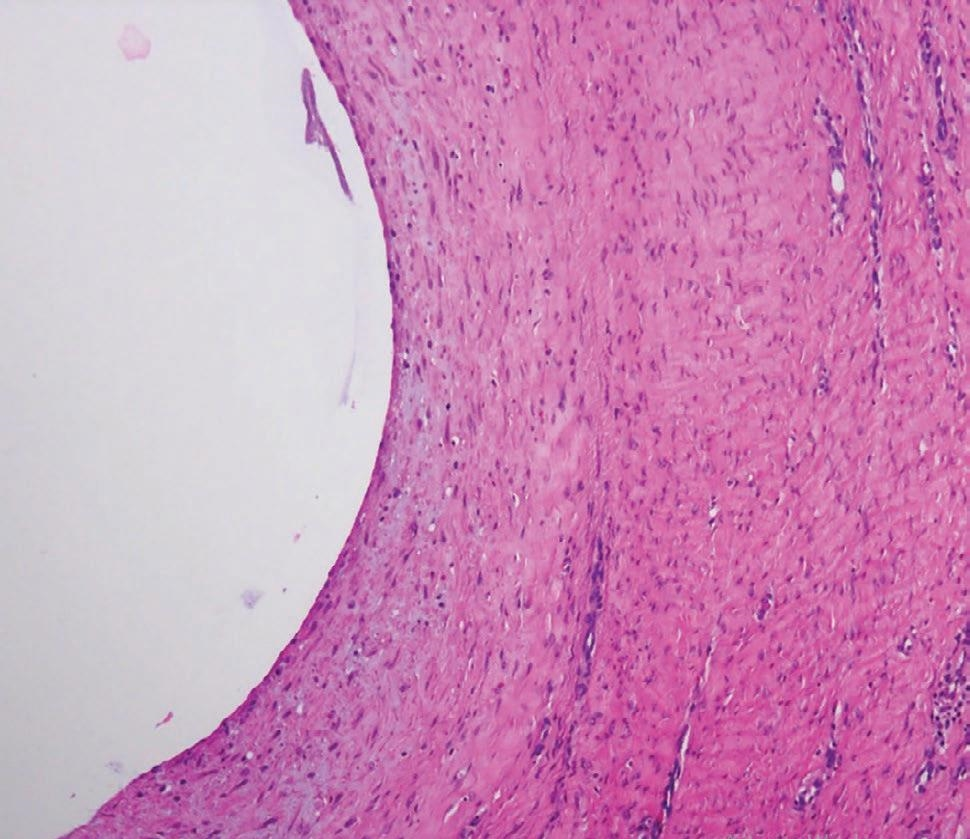
(b)

The patient presented to the clinic at 2 weeks follow‐up with a clean and dry wound, full range of motion of the left knee, and no residual mass or discomfort apart from the regular post‐op healing scar. She reported resolution of her previous symptoms and was instructed to follow‐up as needed in clinic. The patient was followed‐up until 1 year postoperatively with full knee ROM (−5° to 120°), no clinical signs of recurrence and having resumed normal daily function. Clinical follow‐up is summarized in Table 2.

**Table 2:** Timeline of clinical follow‐up and outcomes.

Clinical follow‐up date	Outcomes	Range of motion
March 2025, 2 weeks post‐op	Wound clean and dry, no pain except at surgical site	10° to 70°
April 2025, 1 month post‐op	Well‐healed scar, no pain	0° to 80°
July 2025, 6 months post‐op	Well‐healed scar, no sign of recurrence	−5° to 100°
March 2026, 12 months post‐op	No pain and no sign of recurrence	−5° to 120°

## 3. Discussion

This report discloses a case of an intramuscular ganglion cyst of the extensor digitorum longus, a location that has not been previously reported in the literature. This lesion was isolated within the muscle belly and was not found to be extending into the knee joint capsule, which is rare in itself. This cyst′s unusual location and nonspecific presentation often constitute a diagnostic challenge, as it can mimic other benign and malignant soft tissue masses, hence necessitating further diagnostic and therapeutic interventions especially in symptomatic lesions [5].

Ganglion cysts are benign, mucin‐filled soft tissue lesions that commonly arise near joints and tendon sheaths [9]. They are more commonly located on the posterior aspect of the wrist and are often periarticular. Intramuscular ganglion cysts, on the other hand, are rare and uncommon manifestations [10]. The latter cysts can present with a variety of clinical signs and symptoms depending on their sizes, location, and proximity to neurovascular structures [8]. Common symptoms include swelling, pain, and often neurological symptoms if surrounding nerves are compressed due to mass effect [6, 11, 12]; nonetheless, the lesion may be asymptomatic and thus diagnosed incidentally.

Because of the nonspecific nature of the symptoms, clinical assessment alone is often insufficient to establish a clear diagnosis [5]. In fact, a differential diagnosis of soft tissue masses can be quite broad, including lipomas, myxomas, sarcomas, hematomas, and other benign and malignant soft tissue tumors. Given the low prevalence of intramuscular ganglion cysts, they are often not considered on the initial differential diagnosis.

Hence, radiologic differentiation is crucial to guide appropriate management and avoid delayed diagnosis and overtreatment. MRI is the imaging modality of choice; the lesion manifests as a well‐circumscribed, homogeneously hyperintense mass on T2‐weighted sequences, with low signal intensity on T1‐weighted images, and no postcontrast enhancement. These features are typical of a fluid‐filled cystic lesion [5]. Moreover, there was no direct communication with the nearby joints, suggesting a primary intramuscular origin.

Despite imaging, surgical excision and histopathological analysis are essential for definitive diagnosis [13]. Surgical excision remains the treatment of choice, especially when the lesion is symptomatic and/or malignancy cannot be excluded. Complete excision of the cyst, including the fibrous capsule, and removal of the pedicle are essential to minimize the risk of recurrence. Incomplete resection and cyst rupture intraoperatively may increase the likelihood of recurrence [14]. However, a trial of ultrasound‐guided aspiration and prolotherapy was discussed with the patient, but the decision was made to proceed with surgical excision to minimize the risk of recurrence.

The pathogenesis of intramuscular ganglion cysts is not fully understood. Hypotheses include mucinous degeneration of connective tissue within the muscle, possibly triggered by repetitive microtrauma or mechanical stress [12]. There is also the theory of proliferation of pluripotent stem cells triggered by stress/trauma [15]. In our case, histopathological examination revealed a cyst wall composed of dense collagenous tissue without an epithelial or synovial lining, filled with mucinous fluid—findings consistent with a ganglion cyst.

Developing a thorough differential diagnosis for lesions in uncommon anatomical locations is essential. Only a few reports have previously reported on cases of intramuscular ganglion cysts of the lower extremity. They were located in the quadriceps, gastrocnemius, extensor digitorum, peroneus longus, and flexor hallucis brevis [1, 12, 14, 16–19]. These benign structures are ideally treated with surgical excision to rule out a malignancy and are resected en bloc to decrease the risk of recurrence.

## 4. Conclusion

Intramuscular ganglion cysts are rare and underreported in the literature since most of them are asymptomatic. Radiographic findings of cystic lesion without definite solid components or enhancement, although suggestive, may not be sufficient for diagnosis, and surgical excision with histological confirmation remains essential to rule out malignancy and prevent recurrence. Future studies and advanced technologies should help increase our knowledge of this clinical entity and contribute to improving diagnosis and management strategies.

## Author Contributions

All authors contributed to manuscript writing and figures preparation.

## Funding

No funding was received for this manuscript.

## Disclosure

All authors reviewed and approved the manuscript.

## Consent

Both oral and written informed consents were obtained regarding the case study and future publications. The authors certify that they have obtained all appropriate patient consent forms. In the form, the patient has given consent for his/her images and other clinical information to be reported in the journal. The patient understands that his/her names and initials will not be published and due efforts will be made to conceal their identity, but anonymity cannot be guaranteed.

## Conflicts of Interest

The authors declare no conflicts of interest.

## Data Availability

The data that support the findings of this study are available from the corresponding author upon reasonable request.
